# Alterations in Physiological, Biochemical, and Molecular Responses of *Impatiens walleriana* to Drought by Methyl Jasmonate Foliar Application

**DOI:** 10.3390/genes14051072

**Published:** 2023-05-12

**Authors:** Marija Đurić, Angelina Subotić, Ljiljana Prokić, Milana Trifunović-Momčilov, Snežana Milošević

**Affiliations:** 1 Institute for Biological Research “Siniša Stanković”, National Institute of Republic of Serbia, Department for Plant Physiology, University of Belgrade, Bulevar despota Stefana 142, 11060 Belgrade, Serbia; heroina@ibiss.bg.ac.rs (A.S.); milanag@ibiss.bg.ac.rs (M.T.-M.); snezana@ibiss.bg.ac.rs (S.M.); 2 Faculty of Agriculture, University of Belgrade, Nemanjina 6, 11080 Belgrade, Serbia; ljprokic@agrif.bg.ac.rs

**Keywords:** drought, methyl jasmonate, oxidative stress, ABA metabolic genes, aquaporins

## Abstract

Drought stress affects plant growth and development through several mechanisms, including the induction of oxidative stress. To cope with drought, plants have drought tolerance mechanisms at the physiological, biochemical, and molecular levels. In this study, the effects of foliar application of distilled water and methyl jasmonate (MeJA) (5 and 50 µM) on the physiological, biochemical, and molecular responses of *Impatiens walleriana* during two drought regimes (15 and 5% soil water content, SWC) were investigated. The results showed that plant response depended on the concentration of the elicitor and the stress intensity. The highest chlorophyll and carotenoid contents were observed at 5% SWC in plants pre-treated with 50 µM MeJA, while the MeJA did not have a significant effect on the chlorophyll a/b ratio in drought-stressed plants. Drought-induced formation of hydrogen peroxide and malondialdehyde in plants sprayed with distilled water was significantly reduced in plant leaves pretreated with MeJA. The lower total polyphenol content and antioxidant activity of secondary metabolites in MeJA-pretreated plants were observed. The foliar application of MeJA affected the proline content and antioxidant enzyme activities (superoxide dismutase, peroxidase, and catalase) in plants that suffered from drought. The expression of abscisic acid (ABA) metabolic genes (*IwNCED4*, *IwAAO2*, and *IwABA8ox3*) was the most affected in plants sprayed with 50 µM MeJA, while of the four analyzed aquaporin genes (*IwPIP1*;*4*, *IwPIP2*;*2*, *IwPIP2*;*7*, and *IwTIP4*;*1*), the expression of *IwPIP1*;*4* and *IwPIP2*;*7* was strongly induced in drought-stressed plants pre-treated with 50 µM MeJA. The study’s findings demonstrated the significance of MeJA in regulating the gene expression of the ABA metabolic pathway and aquaporins, as well as the considerable alterations in oxidative stress responses of drought-stressed *I. walleriana* foliar sprayed with MeJA. The results improved our understanding of this horticulture plant’s stress physiology and the field of plant hormones’ interaction network in general.

## 1. Introduction

Drought is one of the most important environmental factors affecting plant growth and development worldwide [1]. Disruption of some metabolic processes, including photosynthesis, respiration, transit of carbohydrates, cellular energy reduction, and redox imbalance, decreases plant growth under drought conditions [2]. One of the critical mechanisms limiting plant growth under drought stress is oxidative stress, followed by over-production of reactive oxygen species (ROS) in cells [3,4,5,6]. Under drought, plants are frequently exposed to oxidative stress, leading to membrane lipid peroxidation, as well as nucleic acid and protein damage [7,8]. This is followed by a series of physiological and biochemical changes that can lead in metabolic disorders and ultimately reduce plant growth and yield [9,10]. Drought tolerance in plants is associated with several physiological, biochemical, and molecular alterations. These include changes in pigment composition, osmotic adjustment, secondary metabolism, and antioxidant system defense, as well as changes in gene expression [11,12,13,14,15]. Changes in physiological and biochemical parameters, in turn, lead to re-programming plant metabolism to minimize drought-induced damage. These changes are controlled by molecular mechanisms, namely, by changes in the expression of specific genes [16]. 

Jasmonates (include jasmonic acid—JA, jasmonoyl-isoleucine—JA-Ile, and methyl jasmonate—MeJA) are natural plant growth regulators that play an important role in increasing plant resistance to environmental stresses such as drought, with the most well-known effects being of the JA and MeJA [17,18]. In general, the effects of jasmonates on plants depend on the type of stress, plant species, plant developmental stage, application method, and applied concentration. Many studies suggest that foliar application of jasmonate methylated derivative, MeJA, could enhance plant drought tolerance. In soybean, foliar application of MeJA increased the antioxidant enzyme activities, proline content, and relative water content (RWC) under drought [19]. Similar results were described in *Triticum aestivum* [20], as well as in a citrus species, where the foliar application of MeJA affected the chlorophyll and sugar content [21]. The increased activity of antioxidant defense system and endogenous abscisic acid (ABA) in *Brassica oleracea* was observed after drought exposure of plants pre-treated with MeJA or its functional analog coronatine [22]. Exogenous application of MeJA in maize increased the content of different metabolites, as well as the activity of the antioxidant enzymes [23]. The effect of foliar-applied MeJA on the reducting stomatal conductance and transpiration in wheat and *Vigna unguiculata* was also observed [24,25]. Increased levels of secondary metabolites, such as phenolic compounds, during drought and foliar application of MeJA were also observed in many plant species [26,27,28,29,30]. Thus, MeJA efficiently increases the ability of plants to resist drought by altering various physiological and biochemical responses, such as antioxidant enzyme activity, osmoprotectants, and secondary metabolism. In addition, MeJA can induce the expression of numerous defense genes to mitigate drought stress, as described in wheat [31] and two pearl millet cultivars [32]. In barley, MeJA increased the expression of four tonoplast aquaporin genes under normal growth conditions [33]. Aquaporins form channels in cell membranes and can transport various molecules through them, with water channel function being the best described [34]. Aquaporin expression was affected by drought in *Impatiens walleriana* [35] and by MeJA foliar application in *Setaria italica* [36]. However, it is not well understood how jasmonates affects the expression of aquaporins in different plant species under drought or other abiotic stresses. 

A large number of genes responsible for drought tolerance in plants are influenced by MeJA, and many of them are also positively regulated by ABA, suggesting overlapping signaling pathways between these two hormones. The authors Harb et al. [37] proposed that endogenous jasmonates, in conjunction with high ABA concentration, stimulates the adaptive response of plants in the early stages of drought stress, on the basis of a study with ABA and jasmonate mutants. The same authors hypothesized that the prolonged water deficit reduced the biosynthesis of jasmonates to reduce their inhibitory effect. This hypothesis was confirmed by de Ollas et al. [38], and similar results have been described in rice [39]. Recent research has also used inhibitors of the biosynthesis ABA and JA in wheat, suggesting that JA affects the biosynthesis of ABA and thus the response to drought, whereas ABA does not affect JA biosynthesis [40]. However, there is no evidence on how jasmonates in general affect the expression of genes involved in the ABA metabolic pathway during drought. 

In this work, the effects of foliar-applied MeJA at different concentrations (5 and 50 µM) on the physiological, biochemical, and molecular responses of *I. walleriana* grown ex vitro during drought were investigated. As one of the most popular horticultural plants worldwide, *I. walleriana* has been important in the Serbian horticultural production for many years. As this plant species is drought sensitive, production, transport, and marketing depend on sufficient water supply at all stages. For this reason, studies in our laboratory are aimed at finding new strategies to improve drought tolerance of *I. walleriana*. Salycilic acid had been shown to have a water-deficit-reducing effect on in vitro- and ex vitro-grown *I. walleriana* [41,42]. Moreover, the authors Đurić et al. [43] described the growth-promoting effect of MeJA (5 µM) on physiological and biochemical responses of *I. walleriana* shoots in vitro. It was hypothesized that foliar application of MeJA under ex vitro conditions could also modify the physiological, biochemical, and molecular response of *I. walleriana*, which would enhance the plant’s ability to resist drought. Consistent with this hypothesis, in this study, we examined the changes in pigment composition, proline, total polyphenol content, and antioxidant activity of secondary metabolites, indicators of oxidative stress, and changes in antioxidant enzyme activities. The expression of two genes involved in the biosynthesis of ABA (*IwNCED4* and *IwAAO2*), as well as a catabolic gene (*IwABA8ox3*), was studied in *I. walleriana* in response to MeJA foliar treatment during drought. Proteins encoded by *NCED*, *AAO,* and *ABA8ox3* genes in plants are 9-cis-epoxycarotenoid dioxygenase, abscisic acid aldehyde oxidase, and abscisic acid hydroxylase [44]. The first protein is involved in carotenoid precursor cleavage in the chloroplasts and is considered as the key enzyme in ABA biosynthesis, while the second protein catalyzes the final step in ABA biosynthesis in the cytoplasm. In ABA catabolism, the enzyme hydroxylase commonly hydroxylates ABA at the C-8 position. Interaction between these enzymes is crucial for adequate ABA concentration in plants, depending on physiological conditions (Nambara and Marion Poll, 2005). In addition, the effect of foliar application of MeJA on the expression of aquaporin genes in *I. walleriana* was investigated, and for this purpose, three plasma membrane isoforms (*IwPIP1*;*4*, *IwPIP2*;*2*, and *IwPIP2*;*7*) and one tonoplast isoform (*IwTIP4*;*1*) were analyzed. 

## 2. Results

### 2.1. The Effect of Foliar-Applied MeJA on Photosynthetic Pigment Content in Drought-Stressed I. walleriana

**Figure 1:**
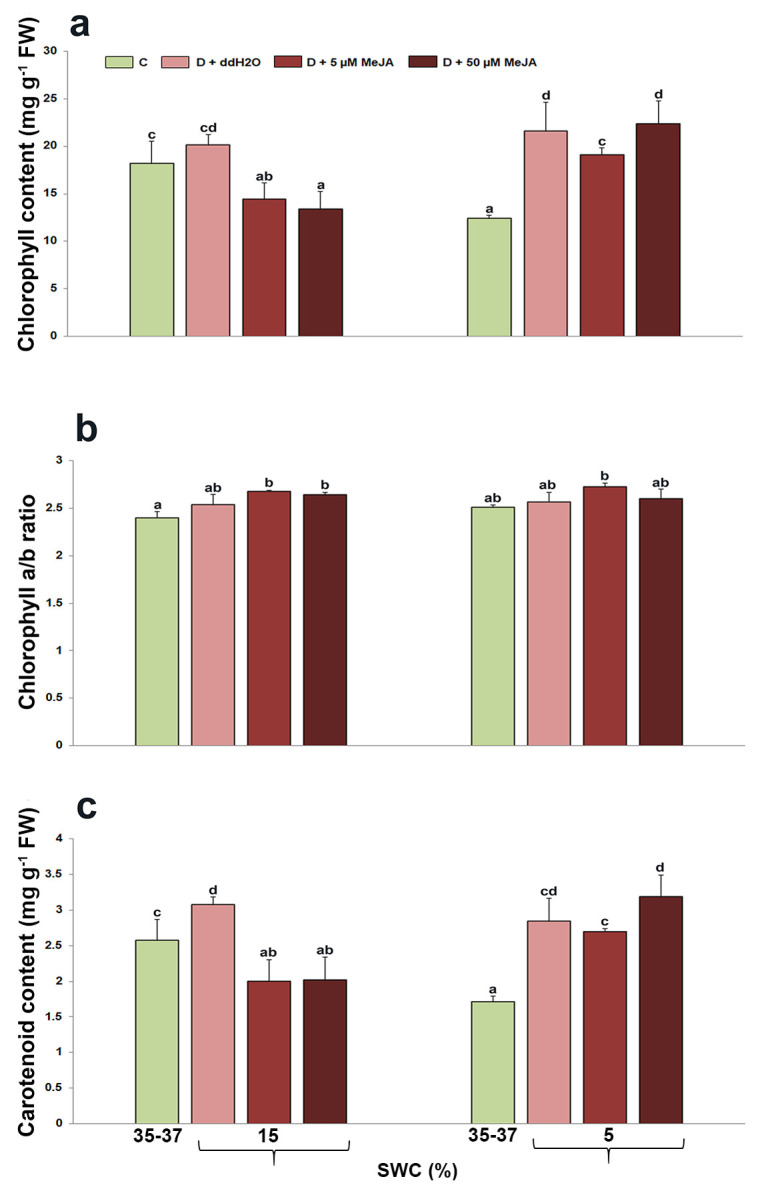
The effect of foliar-applied MeJA on total chlorophyll (**a**), chlorophyll a/b ratio (**b**), and total carotenoid content (**c**) in drought-stressed *I. walleriana* at 15 and 5% SWC. SWC—soil water content; C—control; D + ddH_2_O—drought + distilled water; D + 5 µM MeJA—drought + 5 µM methyl jasmonate; D + 50 µM MeJA—drought + 50 µM methyl jasmonate: FW—fresh weight. Data represent mean ± standard error, with significant differences between treatments based on the LSD test (*p* ≤ 0.05). Different lowercase letters indicate significant differences between treatments.

Photosynthetic pigment content in *I. walleriana* leaves foliar sprayed with MeJA was affected during drought (Figure 1). At 15% soil water content (SWC), total chlorophyll and chlorophyll a/b ratio did not change significantly, while total carotenoid content increased by 19% in drought-stressed plants pre-treated with ddH_2_O, compared with control plants (Figure 1). However, *I. walleriana* leaves pre-treated with both MeJA concentrations (5 and 50 µM) had considerably decreased total chlorophyll (for 28 and 34%, respectively) and carotenoid content (for 35 and 34%, respectively) at 15% SWC, compared with pigment content in *I. walleriana* leaves pre-treated with ddH_2_O. At the same SWC, the chlorophyll a/b ratio was slightly higher in the leaves previously pre-treated with both MeJA concentrations compared with the control, but without significant differences between drought-stressed groups. At 5% SWC, drought-stressed plants foliar sprayed with ddH_2_O increased total chlorophyll and carotenoid content for 74 and 66%, compared with control plants. In *I. walleriana* leaves foliar sprayed with 5 µM MeJA, the total chlorophyll content was lower than in plants foliar sprayed with ddH_2_O. Foliar pre-treatment with 50 µM MeJA caused no significant changes in total carotenoid and chlorophyll content, being similar as in plants foliar sprayed with ddH_2_O at 5% SWC. There were also no significant differences in chlorophyll a/b ratio at 5% between all treatment groups. The interaction between drought and MeJA significantly affected total chlorophyll and carotenoid content (*p* < 0.01), while neither drought nor MeJA had a significant effect on the chlorophyll a/b ratio (*p* = 0.8061, *p* = 0.7779) (Table 1).

### 2.2. The Effect of Foliar-Applied MeJA on Total Polyphenol Content and DPPH Activity in Drought-Stressed I. walleriana

**Figure 2:**
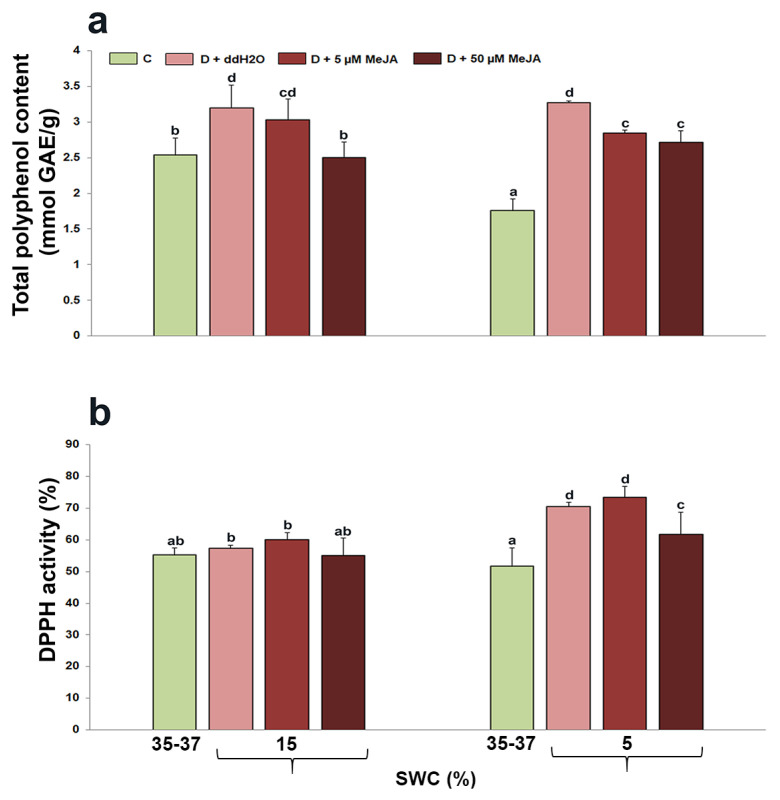
The effect of foliar-applied MeJA on total polyphenol content (**a**) and DPPH activity (**b**) in drought-stressed *I. walleriana* at 15 and 5% SWC. SWC—soil water content; C—control; D + ddH_2_O—drought + distilled water; D + 5 µM MeJA—drought + 5 µM methyl jasmonate; D + 50 µM MeJA—drought + 50 µM methyl jasmonate; GAE—gallic acid. Data represent mean ± standard error, with significant differences between treatments based on the LSD test (*p* ≤ 0.05). Different lowercase letters indicate significant differences between treatments.

On the basis of the statistical analysis, total polyphenol content and DPPH activity were found to be affected by drought and interaction with MeJA (*p* < 0.05) (Table 1). The changes in total polyphenol content and DPPH activity in drought-stressed *I. walleriana* foliar sprayed with different MeJA concentrations are presented in Figure 2. A SWC of 15% resulted in an increase in total polyphenol content for 26% in plants sprayed with ddH_2_O, while there were no significant changes in DPPH content. However, plants foliar sprayed with 50 µM MeJA had lower total polyphenol content for 22%, compared with drought-stressed plants foliar sprayed with ddH_2_O (values similar as in control), while MeJA did not significantly affect DPPH activity. Soil water content of 5% caused the highest increment in total polyphenol content (by 86%) in plants foliar sprayed with ddH_2_O, compared with control plants. On contrary, in plants foliar sprayed with 5 and 50 µM MeJA, total polyphenol content was significantly lower. In comparison to control plants, DPPH activity increased for 37% in plants foliar sprayed with ddH_2_O at 5% SWC. There were no significant changes between these and drought-stressed plants pre-treated with 5 µM MeJA, while plants foliar sprayed with 50 µM MeJA had reduced DPPH activity compared with these two treatment groups. It was noted that this reduced DPPH activity was still significantly higher in comparison to control plants.

### 2.3. The Effect of Foliar-Applied MeJA on Proline Content

**Figure 3:**
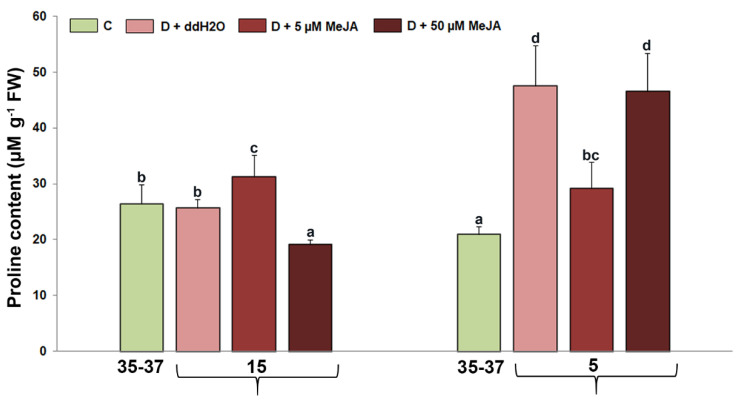
The effect of foliar-applied MeJA on proline content in drought-stressed *I. walleriana* at 15 and 5% SWC. SWC—soil water content; C—control; D + ddH_2_O—drought + distilled water; D + 5 µM MeJA—drought + 5 µM methyl jasmonate; D + 50 µM MeJA—drought + 50 µM methyl jasmonate; FW—fresh weight. Data represent mean ± standard error, with significant differences between treatments based on the LSD test (*p* ≤ 0.05). Different lowercase letters indicate significant differences between treatments.

Figure 3 shows the changes in proline content in drought-stressed *I. walleriana* sprayed with MeJA, and the ANOVA showed that proline content was significantly affected by drought and the drought + MeJA interaction (*p* < 0.05) (Table 1). At 15% SWC, the highest proline increment was observed in plants pre-treated with 5 µM MeJA (for about 20% compared to drought-stressed plants, sprayed with ddH_2_O), while at the same point, in plants foliar sprayed with 50 µM MeJA, proline content was the lowest. In contrast, during intensive drought caused by substrate irrigation to 5% SWC, plants pre-treated with ddH_2_O and 50 µM MeJA had the highest proline content (increment about 120%, compared with control plants). However, there was no significant differences between these two drought-stressed groups, while in plants foliar sprayed with 5 µM MeJA, proline content was lower. 

### 2.4. The Effect of Foliar-Applied MeJA on Hydrogen Peroxide and Malondialdehyde Content

**Figure 4:**
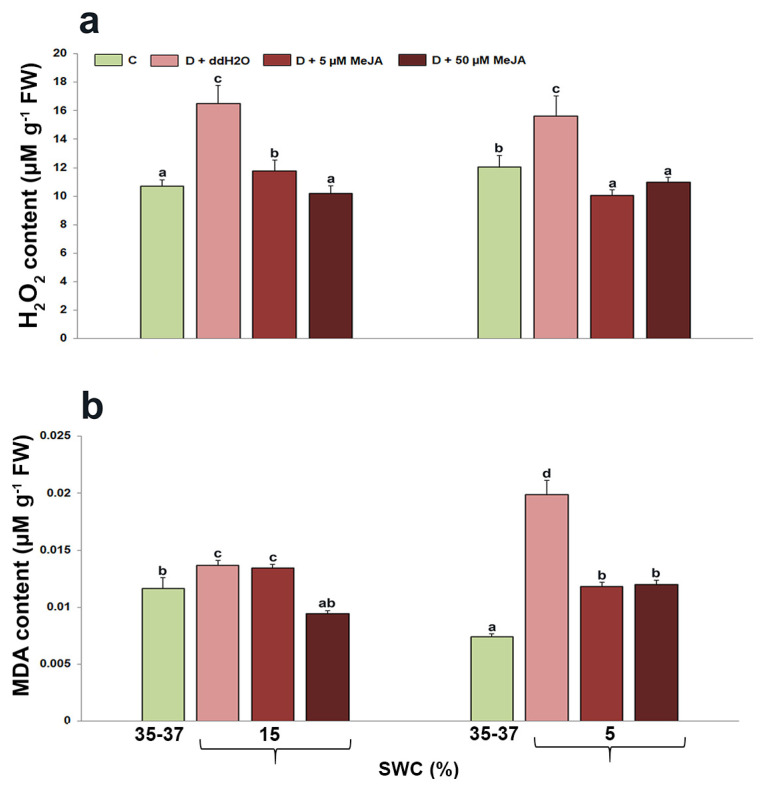
The effect of foliar-applied MeJA on H_2_O_2_ (**a**) and MDA (**b**) content in drought-stressed *I. walleriana* at 15 and 5% SWC. SWC—soil water content; C—control; D + ddH_2_O—drought + distilled water; D + 5 µM MeJA—drought + 5 µM methyl jasmonate; D + 50 µM MeJA—drought + 50 µM methyl jasmonate; FW—fresh weight. Data represent mean ± standard error, with significant differences between treatments based on the LSD test (*p* ≤ 0.05). Different lowercase letters indicate significant differences between treatments.

Oxidative stress indicators—H_2_O_2_ and MDA content—were significantly changed in *I. walleriana* leaves that were foliar pre-treated with MeJA and exposed to drought (*p* < 0.05) (Figure 4). At both drought intensities (15 and 5% SWC), H_2_O_2_ content significantly increased in plants foliar sprayed with ddH_2_O (by 54 and 30%, respectively), compared with control plants (*p* < 0.01). On the other hand, H_2_O_2_ content was significantly lower in plants foliar sprayed with both MeJA concentrations at 15 and 5% SWC in comparison to plants foliar sprayed with ddH_2_O. Compared with control plants, MDA content was significantly higher in drought-stressed plants sprayed with ddH_2_O, and then irrigated to 15 and 5% (by 17 and 167%, respectively, *p* < 0.01). However, plants pre-treated with 50 µM MeJA had the lowest MDA content at 15% SWC (similar to control plants), while plants pre-treated with both MeJA concentrations (5 and 50 µM) had significantly lower MDA content at 5% SWC compared with drought-stressed plants pre-treated with ddH_2_O, but still significantly higher than MDA content in control plants.

### 2.5. The Effect of Foliar-Applied MeJA on Antioxidant Enzyme Activities

**Figure 5:**
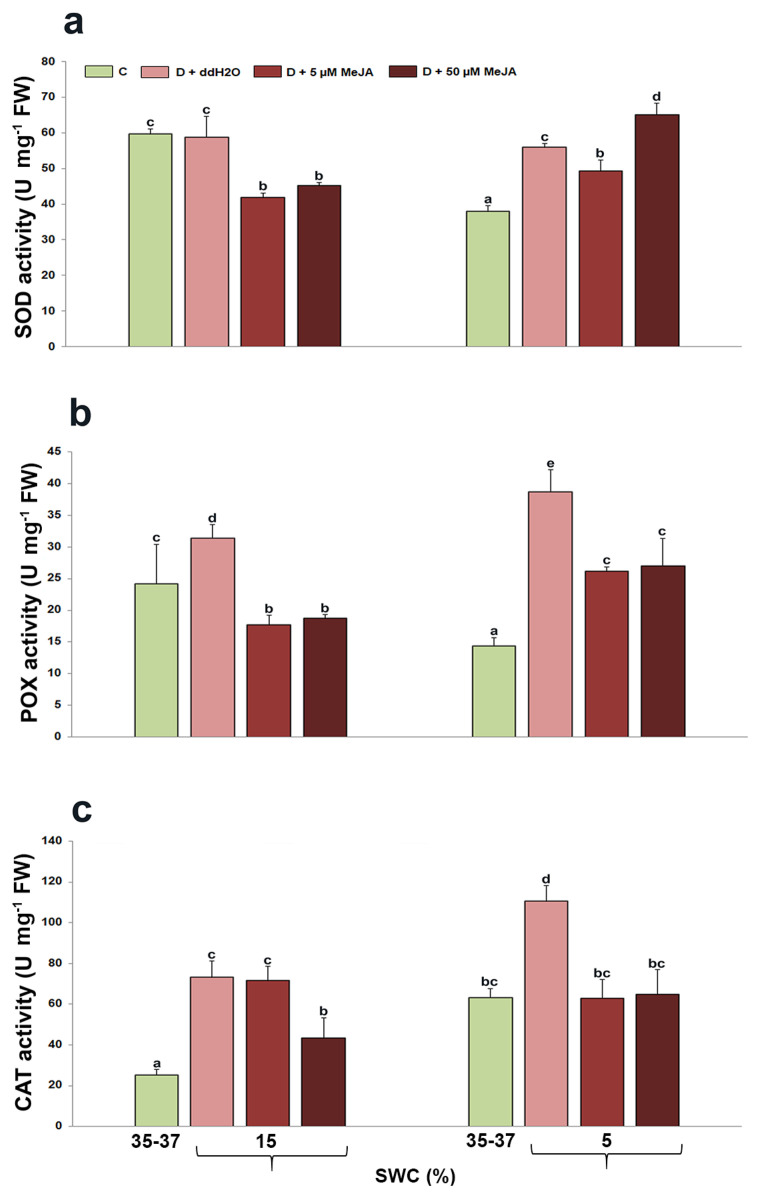
The effect of foliar-applied MeJA on SOD (**a**), POX (**b**), and CAT (**c**) activities in drought-stressed *I. walleriana* at 15 and 5% SWC. SWC—soil water content; C—control; D + ddH_2_O—drought + distilled water; D + 5 µM MeJA—drought + 5 µM methyl jasmonate; D + 50 µM MeJA—drought + 50 µM methyl jasmonate; FW—fresh weight. Data represent mean ± standard error, with significant differences between treatments based on the LSD test (*p* ≤ 0.05). Different lowercase letters indicate significant differences between treatments.

The changes in antioxidant enzyme activities—SOD, POX, and CAT—were evaluated in *I. walleriana* leaves foliar sprayed with MeJA and exposed to drought; this is presented in Figure 5. In plants foliar sprayed with 5 and 50 µM MeJA, SOD activity was significantly lower compared to the control and drought-stressed plants pre-treated with ddH_2_O at 15% SWC (Figure 5a). At 5% SWC, SOD activity was higher in drought-stressed plants foliar sprayed with ddH_2_O, as well as in 5 and 50 µM MeJA compared with control plants (by 47, 30, and 72%, respectively). SOD activity was significantly affected by drought and interaction between drought and MeJA (*p* < 0.01 for both factor) (Table 1).

*I. walleriana* foliar sprayed with ddH_2_O increased POX activity by 30 and 169% at 15 and 5% SWC, respectively, compared with control plants (Figure 5b). Plants sprayed with 5 and 50 µM MeJA, however, showed no significant differences between the two applied drought treatments but exhibited lower POX activity than plants sprayed with ddH_2_O. Statistical analysis showed a significant effect of drought (*p* < 0.05) on POX activity, as well as on drought + MeJA interaction (*p* < 0.05) (Table 1).

At 15 and 5% SWC, CAT activity increased by 190 and 75%, respectively, in drought-stressed plants foliar sprayed with ddH_2_O compared with control plants (Figure 5c). In plants foliar sprayed with 50 µM MeJA, CAT activity was lower at about 40% in comparison to both drought-stressed plants foliar sprayed with ddH_2_O or 5 µM MeJA at 15% SWC. There were no statistically significant changes in CAT activity between drought-stressed plants foliar sprayed with 5 or 50 µM MeJA and control plants at 5% SWC. Similar to POX activity, statistical analysis showed a significant effect of drought and drought + MeJA interaction on CAT activity (*p* < 0.01 and *p* < 0.05, respectively) (Table 1).

### 2.6. The Effect of Foliar Applied MeJA on ABA Metabolic Gene Expression

**Figure 6:**
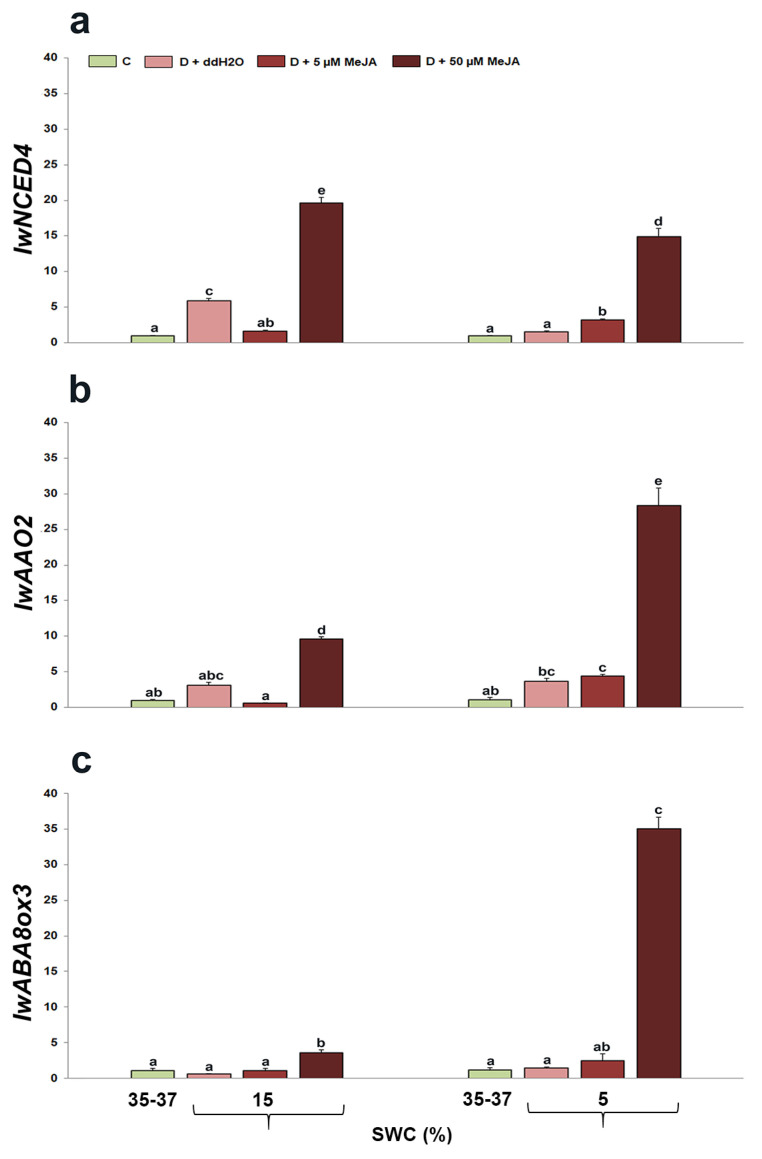
The effect of foliar-applied MeJA on *IwNCED4* (**a**), *IwAAO2* (**b**), and *IwABA8ox3* (**c**) relative gene expression in drought-stressed *I. walleriana* at 15 and 5% SWC. SWC—soil water content; C—control; D + ddH_2_O—drought + distilled water; D + 5 µM MeJA—drought + 5 µM methyl jasmonate; D + 50 µM MeJA—drought + 50 µM methyl jasmonate. Data represent mean ± standard error, with significant differences between treatments based on the LSD test (*p* ≤ 0.05). Different lowercase letters indicate significant differences between treatments.

Drought and the drought + MeJA interaction significantly affected *IwNCED4* (*p* < 0.001 for both factors), as well as *IwAAO2* (*p* < 0.001) and *IwABA8ox3* (*p* < 0.001) gene expression. The changes in ABA metabolic gene expression (*IwNCED4*, *IwAAO2*, and *IwABA8ox3*) are presented in Figure 6. On the basis of the obtained results, it can be noticed that plants foliar sprayed with 50 µM MeJA had the highest relative expression of all ABA metabolic genes (*IwNCED4*, *IwAAO2*, and *IwABA8ox3*) at both drought intensities compared with control plants. Plants foliar sprayed with ddH_2_O increased *IwNCED4* expression at 15% SWC, while they did not have a significant effect on *IwAAO2* and *IwABA8ox3* expression at both 15 and 5% SWC compared with control plants. Plants foliar sprayed with 5 µM MeJA increased *IwNCED4* expression at 5% SWC and *IwAAO2* expression at 5% SWC compared with control plants.

### 2.7. The Effect of Foliar-Applied MeJA on Aquaporin Gene Expression

**Figure 7:**
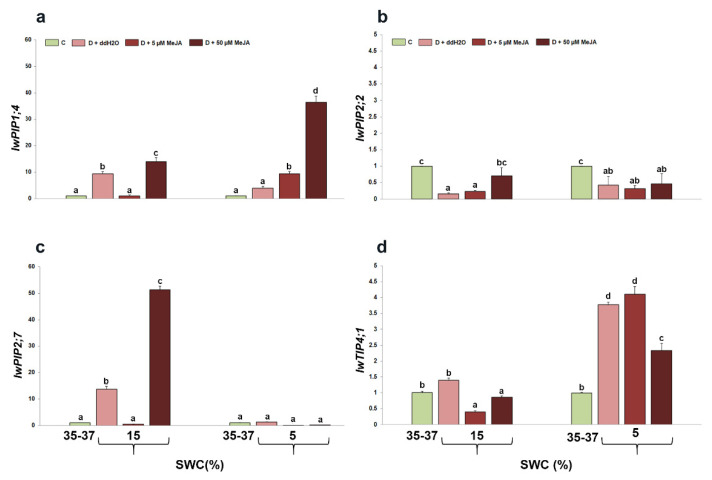
The effect of foliar-applied MeJA on *IwPIP1*;*4* (**a**), *IwPIP2*;*2* (**b**), *IwPIP2*;*7* (**c**), and *IwTIP4*;*1* (**d**) relative gene expression in drought-stressed *I. walleriana* at 15 and 5% SWC. SWC—soil water content; C—control; D + ddH_2_O—drought + distilled water; D + 5 µM MeJA—drought + 5 µM methyl jasmonate; D + 50 µM MeJA—drought + 50 µM methyl jasmonate. Data represent mean ± standard error, with significant differences between treatments based on the LSD test (*p* ≤ 0.05). Different lowercase letters indicate significant differences between treatments.

The relative expression of *IwPIP1*;*4* was significantly affected by drought (*p* < 0.001) and the drought + MeJA interaction (*p* < 0.001). Relative expression of *IwPIP1*;*4* was strongly induced in plants foliar sprayed with ddH_2_O and 50 µM MeJA at 15% SWC compared with control plants. However, at 5% SWC, *IwPIP1*;*4* expression increased in both MeJA foliar-treated plant groups, with the greatest increment observed in plants foliar sprayed with 50 µM MeJA (Figure 7a). The expression of *IwPIP2*;*2* showed more moderate changes and was reduced in all treated plant groups during drought, compared with the control plants (Figure 7b). However, among the drought-stressed plants, only plants foliar sprayed with 50 µM MeJA showed a slight increment at 15% SWC. On the basis of the statistical analysis, there were no observed significant effects of drought (*p* = 0.5784) and the interaction between drought and MeJA treatment (*p* = 0.4181) on *IwPIP2*;*2* expression. The expression pattern of *IwPIP2*;*7* was similar to the *IwPIP1*;*4* expression pattern at 15% SWC (Figure 7c). Namely, plants foliar sprayed with ddH_2_O increased *IwPIP2*;*7* expression at 15% SWC, while the expression was the highest in plants foliar sprayed with 50 µM MeJA. Accordingly, significant effects of drought (*p* < 0.001) and drought + MeJA interactions (*p* < 0.001) were observed. At 5% SWC, there were no significant changes in *IwPIP2*;*7* expression between drought-stressed and control plants. Similar to changes in the expression of *IwPIP2*;*2*, the expression pattern of *IwTIP4*;*1* showed moderate changes (Figure 7d). At 15% SWC, *IwTIP4*;*1* was reduced in plants foliar sprayed with 5 and 50 µM MeJA, compared with control and drought-stressed plants foliar sprayed with ddH_2_O. At 5% SWC, *IwTIP4*;*1* expression was induced in all drought-stressed plants, with the highest increment observed in plants foliar sprayed with ddH_2_O and 5 µM MeJA, compared to control plants. Both drought and drought + MeJA interaction significantly affected *IwTIP4*;*1* expression (*p* < 0.001).

## 3. Discussion

### 3.1. Physiological and Biochemical Responses of I. walleriana to Drought and MeJA

*I. walleriana* leaves’ photosynthetic pigments changed in response to drought and MeJA foliar application. Chlorophylls and particularly carotenoids may act as antioxidants [45], and the reduced content after MeJA treatments in drought-stressed *I. walleriana* could indicate that these plants potentially have lower levels of the oxidative stress by-products. This means that elicitor MeJA could act in way to reduce oxidative stress by-products by activating defense response [19,46], which could be reflected in lower level of other antioxidants (such as pigments), since there is no need for their action. Lower total chlorophyll and carotenoid content were also observed in sweet potato treated with MeJA during desiccation [30]. Since there were no differences in chlorophyll a/b ratio between different drought-stressed foliar-treated groups, it could be concluded that MeJA did not have an impact on the ratio between chlorophyll a and b. At 5% SWC, total chlorophyll and carotenoid content increased in all treated groups compared with control plants, with the highest carotenoid content in plants pre-treated with 50 µM MeJA. These responses indicated that intensive drought differently regulates pigment concentration in *I. walleriana* leaves, which is probably connected with their antioxidant activities. Increased carotenoid content in *I. walleriana* leaves foliar treated with 50 µM MeJA and exposed to intensive drought could be related with their antioxidant activities since the stress was intensified. On the other hand, the reason for increased chlorophyll during dehydration may be mediated by increased endogenous cytokinin accumulation. However, in this study, endogenous cytokinins were not quantified, but a few other studies indicated the relationship between chlorophyll and these plant hormones. According to this, the authors Hassanein et al. [47] described increased endogenous cytokinin content during dehydration in soybean foliar treated with 100 µM MeJA. Contrarily, the authors Ananieva et al. [48] described the reduction of endogenous cytokinin content and chlorophyll content, as well as the accelerated senescence in *Cucurbita pepo* during the exogenous application of MeJA under optimal physical conditions. It is known that the phytohormones cytokinins play a crucial role in chloroplast development and chlorophyll biosynthesis [49].

Total polyphenol content and DPPH activity were affected by MeJA foliar application in drought-stressed *I. walleriana*. Plant responses depended on stress intensity and applied MeJA concentration. At both drought intensities (15 and 5% SWC), total polyphenol content was higher in plants pre-treated with ddH_2_O compared with plants pre-treated with MeJA, which was similar to the changes in MDA and H_2_O_2_ content in *I. walleriana* leaves. Plants pre-treated with 50 µM had the lowest total polyphenol content at 15% SWC. These results unequivocally indicate the effect of MeJA elicitation on prevention of the ROS production and damage products caused by ROS forms, which resulted as reduced accumulation of phenol components during drought. Similar results are described in the medicinal important plant *Dracocephalum kotschyi* (50). Namely, elicitation with 0.5 mM MeJA decreased the total content of polyphenols and antioxidant capacity of plants during dehydration. Moreover, in these plants, MDA and H_2_O_2_ concentrations were also reduced compared to untreated plants exposed to dehydration. The reason for the reduced concentration of these compounds, as well as phenolic components and antioxidant capacity, is by direct neutralization of ROS forms by MeJA, as the authors explain, and therefore plants have no need for biosynthesis of phenolic compounds and activation of the antioxidant defense system. The antioxidant capacity of secondary metabolites measured by the reduction of DPPH radicals indicated that plants increased antioxidant capacity during drought, with the highest recorded values in plants previously pre-treated with ddH_2_O at 5% SWC. Plants previously pre-treated with 50 µM MeJA decreased antioxidant capacities at 5% SWC, and this could be explained as being similar to the results for total polyphenol content, indicating that plants have no need for activation of antioxidants since MeJA contributed to the reduction of oxidative stress. Lower total antioxidant activity (TAA) measured by DPPH and FRAP methods was also described in drought-stressed *D. kotschyi* treated with MeJA [50]. Additionally, there are reports for increased total polyphenol content and antioxidant activity upon MeJA application, indicating that in different plants, MeJA acts in different ways. The effect of MeJA on increased flavonoids and phenolic components was recorded in two soybean cultivars during drought compared with untreated plants [51]. However, the more tolerant soybean cultivar had a lower content of phenolic components in the shoots than a more sensitive cultivar, which to a certain extent can be connected with the results obtained in this research. The foliar application of 50 µM MeJA in *Mentha × piperita* affected the increased content of total polyphenols and flavonoids, both in the control group and in plants exposed to moderate and pronounced water deficit [52]. The addition of MeJA in soybean cell cultures increased the content of total phenolic compounds (flavonoids and isoflavones), which also induced the expression of genes involved in the phenolic biosynthetic pathway [53]. 

The results obtained for proline indicate that proline content in *I. walleriana* leaves during drought was dependent on the elicitor concentration and drought intensity. The accumulation of osmoprotectants, including proline, can reduce the water potential in plant cells, thus preventing water loss and damage to membrane integrity during drought. In plants exposed to substrate irrigation up to 15%, different responses in proline accumulation could be related with the MeJA concentration effect. Increment in proline concentration in plants treated with 5 µM MeJA could contribute to osmoprotection as well in ROS neutralization, since proline also has an antioxidant role [54,55]. On the other hand, decrement in proline accumulation in plants foliar sprayed with 50 µM MeJA (similar to the results for total polyphenol content) could be connected with a strong effect of higher MeJA concentration to ROS neutralization and consequently lower accumulation of antioxidants. According to the obtained results, it could be ascertained that foliar application of MeJA in intensive drought did not have a significant effect on proline accumulation since the accumulation was lower in plants foliar sprayed with 5 µM MeJA compared to plants pre-treated with ddH_2_O, and also no differences were observed between plants foliar treated with ddH_2_O and 50 µM MeJA. In *Beta vulgaris*, foliar-applied MeJA increased proline content during dehydration, as well as the relative water content in cells [56], indicating that the accumulation of osmoprotectants affected the prevention of water loss. However, in these plants, lower accumulation of proline was detected after 4 days of water and strongly increased after 6 days without irrigation. These results indicate that the severity of stress significantly affects proline accumulation, which is similar to in our work, since intensive drought affected proline accumulation the most. Betaine accumulation in *B. vulgaris* was unaffected by any MeJA treatment in drought-stressed plants, in contrast to proline. Such results indicate that different osmoprotectants respond to MeJA during drought, and these results could be related with the result obtained in our study. Namely, instead MeJA did not significantly affect proline accumulation in *I. walleriana* leaves exposed to 5% SWC, potentially could have affected some other osmoprotectants. The effect of MeJA on increasing proline content during dehydration has also been described in *Hordeum vulgare* [57], *V. unguiculata* [25], *Citrus cultivars* [21], and many other plant species. 

Exposure of *I. walleriana* to drought significantly increased the H_2_O_2_ and MDA content in the leaves. It was observed that increased oxidative stress indicators were the highest in drought-stressed plants previously pre-treated with ddH_2_O, compared with plants pre-treated with MeJA. In particular, plants pre-treated with MeJA had much lower levels of H_2_O_2_ and MDA in leaves, compared with plants pre-treated with ddH_2_O. The obtained results indicated the significance of MeJA in reducing the harmful effects of drought by eliminating the oxidative stress by-products. The effect of exogenously applied MeJA on H_2_O_2_ and MDA decrement during drought was also described in *Glycine max* [19], *B. oleracea* [22], *B. napus* [58], *Brassica* sp. [59], *V. unguiculata* [25], *Citrus* cultivar [21], *Phaseolus vulgaris* [60], and many other plant species. However, the opposite effects of MeJA on oxidative stress indicators were also recorded during drought. Namely, in the medicinally important plant *Verbascum sinuatum*, the application of MeJA increased MDA and H_2_O_2_ contents as well as POX activity during drought [61]. Similar results were recently described in *V. nuducuale* [62], where MDA and H_2_O_2_ contents increased, in parallel with increased phenolic compounds. 

On the basis of the antioxidant enzyme analysis in *I. walleriana* leaves, a similar trend in changes as for MDA and H_2_O_2_ contents were observed during drought. At 15% SWC, SOD, POX, and CAT activities were the highest in *I. walleriana* plants pre-treated with ddH_2_O, where the contents of MDA and H_2_O_2_ were also the highest. In the plants previously pre-treated with different MeJA concentrations, the activities of the analyzed enzymes were lower, as well as MDA and H_2_O_2_ contents. On this basis, it can be concluded that the application of MeJA probably reduced the production of ROS as well as the damage products of ROS, which was also reflected as changed activity of antioxidant enzymes during drought in *I. walleriana* leaves, namely, their reduction. The main role of SOD is neutralization of the superoxide-anion radical and production of H_2_O_2_, which is further subjected to conversion by POX, CAT, and other antioxidant enzymes [63]. In plants foliar sprayed with ddH_2_O, both POX and CAT probably had a significant role in elimination products from SOD catalytic reactions at 5% SWC. However, in plants foliar sprayed with MeJA, it seems that POX activity probably had the main role in elimination of ROS produced by SOD, since CAT activity was not different from the control in these plants. This indicates that drought in combination with MeJA differently affected the activities of these two enzymes. Besides this, POX activity was still lower than in plants foliar sprayed with ddH_2_O, indicating that MeJA also in some way reduced its activity. This could be explained as MeJA’s contribution to lower oxidative stress reflected on lower activities of some antioxidant enzymes, but also maybe that MeJA affected increment in some others, such as enzymes involved in the ascorbate–gluthation cycle, which are also very important in eliminating H_2_O_2_ in different cell compartments [63]. In salinity stress, for example, MeJA mainly affected the activity of ascorbat peroxidase (APX), while POD and SOD remained unchanged in both rice cultivars [64].

The effect of MeJA on antioxidant enzyme activities has also been reported in many plant species. In soybean, foliar application of 50 µM MeJA increased SOD, POX, and CAT activities during drought, which was accompanied by decreased lipid peroxidation [19]. Moreover, in cabbage, the application of MeJA and coronatin, a MeJA functional analog, affected increased antioxidant enzyme activities during drought and reduced MDA and H_2_O_2_ contents (22). In barley, foliar application of 100 µM MeJA increased POX and ascorbate peroxidase (APX) activities during drought, but decreased CAT activity. In these plants, the contents of MDA and H_2_O_2_ during drought were increased, which also explains the increased activity of antioxidant enzymes [65]. The use of jasmonic acid in *Pennisetum glaucum* during drought induced by polyethylene glycol in vitro increased the activities of antioxidant enzymes (SOD, APX, and CAT) and decreased the content of MDA and H_2_O_2_ [66]. In *P. vulgaris* treated with MeJA, increased SOD activity was noted, whereas CAT activity was unchanged, and POX activity was slightly increased, compared with untreated plants exposed to drought [60]. Compared with untreated plants exposed to drought, MeJA affected an increase in enzymes involved in the ascorbate–glutathione cycle, indicating the importance of the ascorbate–glutathione cycle in the elimination of ROS forms. Depending on the genotype, type of stress, and applied elicitor, different enzymatic and non-enzymatic components could play a dominant role in the elimination of ROS.

### 3.2. Molecular Responses of I. walleriana to Drought and MeJA

Stomatal closure during drought prevents excessive water loss through transpiration. Plants reduce stomatal conductance during drought, thus providing higher water retention in tissues and higher cell turgidity. Such results were recorded in different cultivars of *V. vinifera* [67,68], *Cicer arietinum* [69], *Oryza sativa* [70,71], *Zea mays* [72], and many other plant species. Increased ABA concentration during drought affected stomatal closure by regulating turgor and the ion contents in guard cells. The changes in ABA concentration during drought stress are caused by changes in ABA metabolism and genes involved in ABA biosynthesis and catabolism. Previously, the authors Đurić et al. [73] described the increment in ABA content in drought-stressed *I. walleriana*, as well as changes in the expression of ABA metabolic genes. Similarly, drought-stressed *P. vulgaris* genotypes increased expression of *NCED* genes, which control the first step in ABA biosynthesis, with a higher expression level in the tolerant rather than the susceptible genotypes [74]. Similar results were described for carrot cultivars [75], *Fagopyrum esculentum* [76], and *Hevea brasiliensis* [77]. In this work, we showed that *I. walleriana* foliar sprayed with 50 µM MeJA had the highest expression of all analyzed ABA metabolic genes—*IwNCED4*, *IwAAO2*, and *IwABA8ox3*—during both drought intensities (15 and 5% SWC). The reason for increased expression of the catabolic gene *IwABA8ox3* in plants pre-treated with a higher MeJA concentration during drought may be explained by the increased expression of biosynthetic genes *IwNCED4* and *IwAAO2*, i.e., positive feedback. A similar explanation was provided by the authors Cai et al. [78] for the upregulation of the rice *OsABA8ox3* gene during drought, which could be the result of the feedback regulation caused by the increase in ABA content and *OsNCED3* expression. It is also suggested that *OsABA8ox3* is the main ABA catabolic gene that negatively regulates the ABA level under drought stress. Namely, transgenic plants with silenced *OsABA8ox3* were more drought tolerant, while plants with *OsABA8ox3* overexpression were hypersensitive to drought. As a result, the authors concluded that the *OsABA8ox3* gene is crucial for regulating ABA level and drought stress tolerance in rice. However, at 15% SWC, the expression level of biosynthetic genes in *I. walleriana* was higher than the catabolic gene, while in intensive drought, the catabolic gene expression increased significantly in plants pre-treated with 50 µM MeJA and was higher than the expression of both biosynthetic genes. The results described in this study suggest that the jasmonate and ABA signaling pathways work together to control each other’s drought responses and that there is a possible mutual exclusion between these two hormones depending on stress intensity. Namely, during the intensive drought (5% SWC), jasmonate could play a dominant role in the responses of *I. walleriana* to drought since the ABA catabolism is favored, while at moderate drought stress (15% SWC), ABA could play a main role since the biosynthesis is favored. Opposite to this, the downregulation of jasmonate biosynthesis and signaling pathways in prolonged drought conditions in *Arabidopsis thaliana* [37] indicate that interaction between hormones is also genotype dependent. Plants previously pre-treated with ddH_2_O manifested increased ABA biosynthesis gene *IwNCED4* at 15% SWC, while the catabolic gene expression was not changed in relation to control plants, pointing out the ABA biosynthesis prevalence during drought. Plants previously pre-treated with 5 µM MeJA increased the expression of *IwNCED4* and *IwAAO2* more significantly at 5% SWC, while at the same time, the expression of the *IwABA8ox3* gene was lower. Such results indicate that plant response is dependent on applied elicitor concentration and stress intensity. Moreover, in this case, it is possible that the jasmonate concentration was higher at the beginning of the stress period, and that ABA accumulation prevailed during more intensive drought.

On the basis of the analysis of aquaporin gene expression in *I. walleriana* leaves during drought, it was observed that foliar-sprayed MeJA affected the changes in gene expression depending on applied elicitor concentration and stress intensity. When examining both SWC treatments, it was shown that the greatest effect on *IwPIP1*;*4* increment had a foliar application of 50 µM MeJA. These results could be explained by a better flow of water through the cells to maintain homeostasis and turgidity under stress conditions. The similar explanation could be provided for increment in *IwPIP2*;*7* expression at 15% SWC, especially in plants foliar sprayed with 50 µM MeJA. It is obvious that drought and 50 µM MeJA in combination differently affected the expression of *IwPIP2*;*7* at 15 and 5% SWC, indicating that aquaporin expression is dependent on the stress intensity. Stress intensity could reduce, increase, or not change aquaporin expression, and plants adjust responses in accordance to physiological state. Depending on drought stress duration in the plant *Galega orientalis*, aquaporin expression was also induced and declined [79]. The application of MeJA under optimal conditions also affected the changed expression of six aquaporin genes from the *PIP1* and *PIP2* subgroups in the roots of *P. vulgaris*. Also, depending on the applied MeJA concentration, decreased, increased, or unchanged expression of the analyzed genes were recorded [80]. Cis-regulatory elements for MeJA-induced gene expression were identified in the promoters of *TIP* genes in barley, and MeJA treatment was shown to increase the expression of four *TIP* genes [33]. In two *S. italica* cultivars, five genes (*SiPIP1*;*2*, *SiPIP3*;*1*, *SiSIP1*;*1*, *SiNIP1*;*2*, and *SiTIP2*;*2*) were analyzed during dehydration induced by polyethylene glycol, high temperature, and salinity, as well as the application of growth regulators: ABA, MeJA and salicylic acid [36]. During dehydration, the expression of *SiPIP3*;*1*, *SiSIP1*;*1*, and *SiTIP2*;*2* genes were induced in both cultivars, while the expression of the *SiPIP1*;*2* gene was increased only at the beginning (1 h after stress) in the tolerant cultivar, and then reduced 12 and 24 h after dehydration. The expression of all analyzed genes was increased in tolerant cultivar treated with MeJA, while in sensitive cultivar MeJA increased the expression of all analyzed genes, except *SiPIP1*;*1*. During salinity stress, the effect of MeJA foliar application in *Citrus sinensis* increased the expression of aquaporin genes *PIP1*;*1*, *PIP2*;*3,* and *TIP4*;*1* [81]. A moderate pattern of changed expression was observed for *IwPIP2*;*2* and *IwTIP4*;*1* genes in *I. walleriana* leaves during drought. *IwPIP2*;*2* reduced the expression in all drought-stressed foliar-treated groups, compared with control plants, whereas *IwTIP4*;*1* was slightly induced at 5% SWC in plants foliar sprayed with ddH_2_O and 5 µM MeJA. Under drought stress, the expression of aquaporin genes may be suppressed, which may reduce membrane water permeability, in order to avoid water loss from the cells. Our results demonstrated that different *I. walleriana* aquaporins are involved in response to foliar applied MeJA and drought, and that their role during tissue water loss could be attributed to water preservation or water transport between cells to maintain homeostasis.

## 4. Materials and Methods

### 4.1. Plant Material and Experimental Design

The experimental study started with seeds of the *I. walleriana* Xtreme Scarlet variety (Syngenta). Physical conditions during the seed germination and plant growth were the same as described previously by Đurić et al. [73].

**Figure 8:**
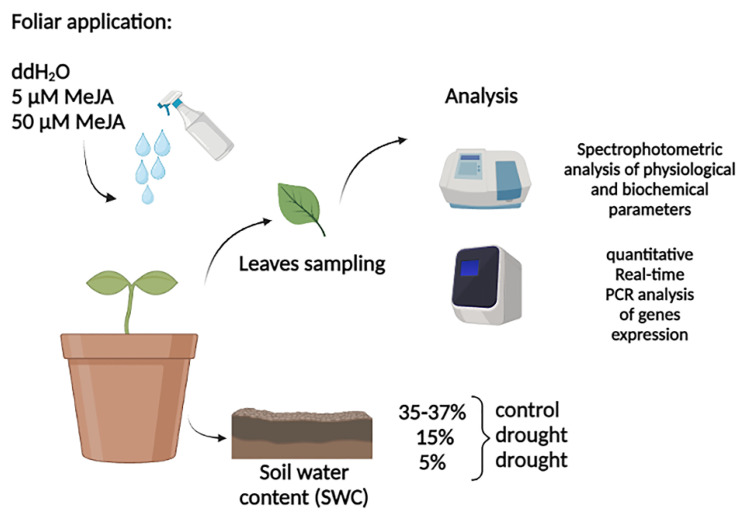
Schematic representation of the experimental design. Foliar spraying of plants was performed using distilled water (ddH_2_O) for control and drought-stressed plants, and methyl jasmonate (MeJA) (5 and 50 µM) for drought-stressed plant groups. Leaves sampling were taken under two different drought regimes, where the soil water content was 15 or 5%, and subsequent analysis were performed.

The experiment with MeJA foliar application included four treatments: control plants pre-treated with distilled water (ddH_2_O) (C), drought-stressed plants pre-treated with ddH_2_O (D + ddH_2_O), drought-stressed plants pre-treated with 5 µM MeJA (D + 5µM MeJA), and drought-stressed plants pre-treated with 50 µM MeJA (D + 50 µM MeJA), with three replicates. Stock solution of MeJA (1M) was made in 96% ethanol, while further dilutions were made with distilled water. In MeJA working solutions (5 and 50 µM), Tween 20 (0.1%) was added additionally, whose detergent properties provide better binding of substances to leaves. In the distilled water used for foliar application, Tween-20 was also added, as well as ethanol, which was used for MeJA stock solution preparation and therefore was present in MeJA working solutions. Foliar spraying with ddH_2_O or MeJA was performed twice, 7 days before drought induction and on the day when drought was imposed. Plants were 44 days old at the time, and they were divided into groups according to their treatment. Depending on treatment, plants were irrigated up to 15 and 5% of soil water content (SWC), while control plants were optimally water supplied through the experimental duration (35–37% of SWC). The leaf samples of the control and drought-stressed plants pre-treated with ddH_2_O, 5 or 50 µM MeJA, were harvested for physiological, biochemical, and molecular analysis at the time when soil moisture levels reached 15 and 5%. To achieve 15% SWC, nine days were needed without water supply, while twenty days were necessary to reach 5% SWC. The leaf samples were frozen in liquid nitrogen and stored at −80 °C until further analysis. A schematic representation of the experiment setup is presented in Figure 8.

### 4.2. Spectrophotometric Analysis of Photosynthetic Pigments

For the photosynthetic pigments analysis, 20 mg of frozen leaf tissue was incubated in a water bath for 10 min at 70 °C with 2 mL of 96% ethanol. The extract was transferred to the cuvettes, and absorbance was measured at three wavelengths: 470, 648, and 664 nm on a UV-Vis spectrophotometer (Agilent 8453, Life Sciences, Santa Clara, CA, USA). Total chlorophyll and carotenoid contents were determined according to the formulas given by Lichtenthaler [82] and expressed as mg g^−1^ of tissue fresh weight (FW).

### 4.3. Spectrophotometric Analysis of the Proline Content

Proline content was determined by a ninhydrin reaction in which the reaction of proline with ninhydrin produces a yellow color, according to a modified method by Bates, Waldren, and Teare [83], described in detail by Trifunović-Momčilov et al. [84]. From 250 mg of leaf tissues, free amino acids were extracted using HPLC-grade methanol. The obtained supernatant was mixed with mixture of 500 μL chloroform and 750 μL HPLC-grade water. The aqueous phase was then separated, extracted again with chloroform, and supernatant evaporated to dryness. The samples were reconstituted in 125 µL of water, and 20 µL of the sample (or proline standard) was combined with 50 µL of the ninhydrin reagent. The samples were heated in a water bath for 4 min at 100 °C, cooled, and then diluted in 930 µL of ethanol. Using reactions with 50 µL of ethanol instead of ninhydrin, which were made in parallel for each sample as blank, the absorbance of the yellow reaction product was measured at 350 nm.

### 4.4. Spectrophotometric Analysis of the Total Polyphenol Content (Folin–Ciocalteu Test) and Antioxidant Activity in Plants (DPPH Method)

The total polyphenol content was determined according to the method described by Singleton et al. [85], while the antioxidant activities of secondary metabolites were determined using the DPPH (1,1′-diphenyl-2-picrylhydrazyl) method according to Brand-Williams et al. [86]. Using the Folin–Ciocalteu test (FC test), plant ethanol extracts were mixed with FC reagent and deionized water at room temperature. After incubation, 20% Na_2_CO_3_ was added into the mixture, which was left at room temperature for 90 min in dark conditions. The following absorbance was measured at 765 nm, while for calculating the total polyphenol content, gallic acid (GAE) was used as a standard phenol. In the DPPH method, plant ethanol extracts were mixed with DPPH reagent solution and methanol. Following incubation at the room temperature, the degree of the DPPH reduction was estimated through the absorbance measurement at 520 nm. More details about the modified Folin–Ciocalteu and DPPH methods used in this work were previously described by the authors Đurić et al. [73].

### 4.5. Spectrophotometric Analysis of the Hydrogen Peroxide and Malondialdehyde Content

For both assays, extraction was accomplished by adding 1.5 mL of 0.1% trichloroacetic acid (TCA) to 150 mg of ground tissue. The hydrogen peroxide (H_2_O_2_) content was measured using the method described by Velikova et al. [87], while the level of lipid peroxidation in leaves was determined by the TBARS method by evaluating the malondialdehyde (MDA) content [88]. In H_2_O_2_ determination, the supernatant was mixed with 10 mM potassium phosphate buffer (pH 7.0) and 1 M potassium iodide (KI). H_2_O_2_ content was determined spectrophotometrically by measuring the absorbance at 390 nm. For MDA evaluation, the supernatant was mixed with 20% TCA and 0.5% 2-thiobarbituric acid (TBA). The reaction mixture was heated at 95 °C for 30 min in water bath and then quickly cooled on ice for five minutes. After centrifugation at 4 °C for 10 min, MDA was determined spectrophotometrically at 532 and 600 nm.

### 4.6. Biochemical Analysis of Antioxidant Enzyme Activities

Extraction of the total soluble proteins was performed as described by Milošević et al. [89], while the content of total soluble proteins was determined by the Bradford method [90]. Total peroxidase (POX) activity was performed spectrophotometrically as described previously by Milošević et al. [89], while total CAT activity was performed according to a slightly modified protocol described by Aebi et al. [91], with details found in the work of Milošević et al. [89]. The activity of superoxide dismutase (SOD) was measured by the modified method of Beyer and Fridovich [92], with details described by Antonić et al. [41].

### 4.7. Analysis of Aquaporin and ABA Metabolic Gene Expression

Isolation of RNA was performed using 100 mg of leaf tissues in a method described by Gašić et al. [93]. After RNA isolation, spectrophotometric measurements (NanoPhotometer^®^ N60, IMPLEN, Munich, Germany) and electrophoresis in denaturing agarose gels showed that the isolated RNA was of high quality and had not deteriorated. Isolated RNA was treated with DNase I (Thermo Fisher Scientific, Waltham, MA, USA) in order to eliminate traces of DNA. Following its use in the reverse transcription reaction (RT-PCR) and the construction of cDNA libraries, RNA quality was evaluated once more. For RT-PCR reactions, we used the commercial kit (Thermo Fisher Scientific, Waltham, MA, USA) according to the manufacturer’s protocol. The obtained cDNA was further used for quantitative real-time PCR (qPCR) analysis. Quantitative real-time PCR was performed for three ABA metabolic (*IwNCED4*, *IwAAO2*, and *IwABA8ox3*) and four aquaporin *(IwPIP1*;*4*, *IwPIP2*;*2*, *IwPIP2*;*7*, and *IwTIP4*;*1*) genes in a QuantStudio 3 Real-Time PCR System (Applied Biosystems). For qPCR analysis, Maxima SYBR Green/Rox qPCR Master Mix (Thermo Fisher Scientific, Waltham, MA, USA) was used in the reaction mixture containing 1 μL of the RT reaction product and the appropriate forward and reverse primers. For qRT-PCR, the following thermal cycling conditions were used: initial denaturation at 95 °C, followed by 40 cycles of denaturation at 95 °C for 30 s, annealing at 60 °C for 30 s, and extension at 72 °C for 30 s. Each sample underwent qRT-PCR in triplicate. Primer sequences and their accession numbers have been described previously in the works of Đurić et al. [35,73].

### 4.8. Statistical Analysis

The STATISTICA software (version 8) was used for ANOVA statistical analysis. The results are shown as means ± standard error (SE). The least significant difference (LSD) test was used to compare the mean differences between experimental treatments, with a statistical significance level of *p* ≤ 0.05. The results were graphically presented using Microsoft Office Excel (2010).

## 5. Conclusions

On the basis of the obtained results, it can be concluded that foliar-applied MeJA differently affected *I. walleriana*’s physiological, biochemical, and molecular responses, depending on drought intensity and applied elicitor concentration. The greatest impact on increased total carotenoid content was observed at 5% SWC in plants pre-treated with 50 µM MeJA. *I. walleriana* foliar sprayed with 50 µM MeJA had the lowest H_2_O_2_, MDA, and total polyphenol content in the leaves at 15% SWC, thus contributing to reducing oxidative stress. At 5% SWC, concentrations of these parameters were similar in both groups pre-treated with 5 and 50 µM MeJA, and lower than in drought-stressed plants foliar sprayed with ddH_2_O, while the DPPH activity was the lowest in plants foliar sprayed with 50 µM. Additionally, during drought, relative SOD, POX, and CAT activities were differently changed. Lower activities of all analyzed enzymes at 15% SWC were observed in plants pre-treated with MeJA compared with plants pre-treated with ddH_2_O, indicating the changes in the level of oxidative stress in plant cells. Moreover, POX and CAT activities at intensive drought were significantly lower in plants foliar sprayed with both MeJA concentrations than in plants foliar sprayed with ddH_2_O. The changes in ABA metabolic genes were affected by MeJA, especially after foliar application of 50 µM MeJA at both drought intensities, indicating the jasmonate involvement in ABA signaling pathway during drought. Foliar-applied MeJA differently affected aquaporin gene expression, with the highest effect on *IwPIP1*;*4* and *IwPIP2*;*7* increment during drought in plants foliar sprayed with 50 µM MeJA. The aquaporin gene expression pattern demonstrated that the MeJA pathway may be involved in the activation of aquaporin genes, whose products are channels for water transport. In general, foliar application of MeJA significantly affected the components of antioxidative system defense and genes involved in the ABA metabolic pathway, as well as aquaporins, thus contributing to plant drought tolerance.

**Figure 9:**
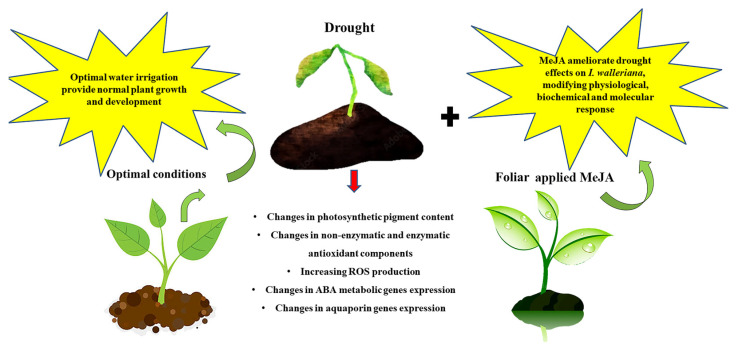
The summarized effects of foliar-applied methyl jasmonate (MeJA) on *I. walleriana* performance under drought. Drought caused alterations at the physiological, biochemical, and molecular levels compared to optimal conditions, which were mitigated by the foliar application of MeJA.

The summarized conclusions are presented in Figure 9.

## Figures and Tables

**Table 1:** Significance of sources of variation (*p* value—probability) after two-way ANOVA analyses for drought, and drought + MeJA interaction effects on all analyzed parameters in the work. Levels of significance: * *p* < 0.05, ** *p* < 0.01, *** *p* < 0.001, ns—no significance.

	Drought	Drought + MeJA
Total chlorophyll content	*p* < 0.01 **	*p* < 0.01 **
Chlorophyll a/b ratio	*p* = 0.8061 ns	*p* = 0.7779 ns
Total carotenoid content	*p* < 0.01 **	*p* < 0.01 **
Total polyphenol content	*p* < 0.05 *	*p* < 0.05 *
DPPH activity	*p* < 0.05 *	*p* < 0.05 *
Proline content	*p* < 0.05 *	*p* < 0.05 *
H_2_O_2_ content	*p* < 0.01 **	*p* < 0.05 *
MDA content	*p* < 0.01 **	*p* < 0.05 *
SOD activity	*p* < 0.01 **	*p* < 0.01 **
CAT activity	*p* < 0.01 **	*p* < 0.05 *
POX activity	*p* < 0.05 *	*p* < 0.05 *
*IwNCED4* expression	*p* < 0.001 ***	*p* < 0.001 ***
*IwAAO2* expression	*p* < 0.001 ***	*p* < 0.001 ***
*IwABA8ox3* expression	*p* < 0.001 ***	*p* < 0.001 ***
*IwPIP1*;*4* expression	*p* < 0.001 ***	*p* < 0.001 ***
*IwPIP2*;*2* expression	*p* = 0.5784 ns	*p* = 0.4181 ns
*IwPIP2*;*7* expression	*p* < 0.001 ***	*p* < 0.001 ***
*IwTIP4*;*1* expression	*p* < 0.001 ***	*p* < 0.001 ***

## Data Availability

All data are included in the manuscript.
